# Determining causes of genetic isolation in a large carnivore (*Ursus americanus*) population to direct contemporary conservation measures

**DOI:** 10.1371/journal.pone.0172319

**Published:** 2017-02-24

**Authors:** Agnès Pelletier, Martyn E. Obbard, Matthew Harnden, Sabine McConnell, Eric J. Howe, Frank G. Burrows, Bradley N. White, Christopher J. Kyle

**Affiliations:** 1 Environmental and Life Sciences Program, Trent University, Peterborough, ON, Canada; 2 Department of Biology, Trent University, Peterborough, ON, Canada; 3 Wildlife Research and Monitoring Section, Ontario Ministry of Natural Resources and Forestry, Peterborough, ON, Canada; 4 Department of Computing and Information Systems, Trent University, Peterborough, ON, Canada; 5 Bruce Peninsula National Park and Fathom Five National Marine Park, Parks Canada, Tobermory, ON, Canada; 6 Forensic Science Department, Trent University, Peterborough, ON, Canada; National Cheng Kung University, TAIWAN

## Abstract

The processes leading to genetic isolation influence a population’s local extinction risk, and should thus be identified before conservation actions are implemented. Natural or human-induced circumstances can result in historical or contemporary barriers to gene flow and/or demographic bottlenecks. Distinguishing between these hypotheses can be achieved by comparing genetic diversity and differentiation in isolated vs. continuous neighboring populations. In Ontario, American black bears (*Ursus americanus*) are continuously distributed, genetically diverse, and exhibit an isolation-by-distance structuring pattern, except on the Bruce Peninsula (BP). To identify the processes that led to the genetic isolation of BP black bears, we modelled various levels of historical and contemporary migration and population size reductions using forward simulations. We compared simulation results with empirical genetic indices from Ontario black bear populations under different levels of geographic isolation, and conducted additional simulations to determine if translocations could help achieve genetic restoration. From a genetic standpoint, conservation concerns for BP black bears are warranted because our results show that: i) a recent demographic bottleneck associated with recently reduced migration best explains the low genetic diversity on the BP; and ii) under sustained isolation, BP black bears could lose between 70% and 80% of their rare alleles within 100 years. Although restoring migration corridors would be the most effective method to enhance long-term genetic diversity and prevent inbreeding, it is unrealistic to expect connectivity to be re-established. Current levels of genetic diversity could be maintained by successfully translocating 10 bears onto the peninsula every 5 years. Such regular translocations may be more practical than landscape restoration, because areas connecting the peninsula to nearby mainland black bear populations have been irreversibly modified by humans, and form strong barriers to movement.

## Introduction

Geographically isolated populations have higher extirpation risks than their contiguous counterparts because they are more likely to experience population size reduction and genetic drift [1–4]. Genetic signatures of population fragmentation and demographic bottlenecks are easily identifiable in the southernmost distribution of many North American carnivores [5–9], because they experienced extreme range contractions [10, 11]. In the northern portion of their range, these carnivores are still continuously distributed, although some populations display reduced genetic variation due to peripherality [12], restriction of movement within preferred habitat [13], or landscape features limiting dispersal [14, 15]. For some of the larger species, preventative management and conservation action may be justified, as they could experience future habitat loss, population size reductions, and genetic isolation in regions of North America that are currently undisturbed [16, 17].

Two elements are needed when establishing whether genetic isolation actually warrants conservation concerns. The first is to estimate baseline genetic diversity and differentiation indices from contiguous populations. The second is to identify drivers of genetic isolation, as some species can survive at extremely low levels of genetic diversity if reduced variability predates demographic bottlenecks [18–21].

Population genetic simulations are powerful tools to identify the processes that lead to genetic isolation and decreased diversity [22–26]. Although many studies now use Bayesian approaches to trace the historical processes that could explain contemporary genetic variation, traditional forward simulations are still useful for understanding the consequences of different environmental and genetic scenarios. Forward simulations can incorporate many variables such as population size, mating system, migration rate, and mutation process [27, 28]. This is particularly important in conservation, as, when ecological and life history traits of species are known, forward simulations can help predict changes in population structure, and assess persistence potential under various biologically realistic scenarios.

Demographic bottlenecks, which may lead to higher local extirpation risks, can be detected via genetic signals such as a heterozygosity excess at polymorphic loci [29, 30], or a decrease in the total number of alleles relative to the range in allele size [31]. In conjunction with forward simulations, the information obtained from these signals helps differentiate between genetic isolation resulting from historical colonization events, which would not hinder persistence on a contemporary timescale [18, 32], and genetic isolation resulting from recent anthropogenic disturbances, which could induce a threat [33].

Among large carnivores, American black bears provide a good model to identify particular situations of genetic isolation that may or may not warrant conservation concerns. First, the species is widely distributed across North America [34–36]. Second, many populations are fragmented and display low levels of genetic diversity that have been attributed to bottlenecks and/or geographic isolation due to habitat loss, fragmentation, and insularity (0.27 < H_E_ < 0.56 [8, 37–40]). Third, the majority of northern populations are highly connected and genetically diverse (0.70 < H_E_ < 0.94 [14, 37, 41, 42]), except for a few genetically distinct populations found in the vicinity of the continuous core [14, 42].

**Fig 1:**
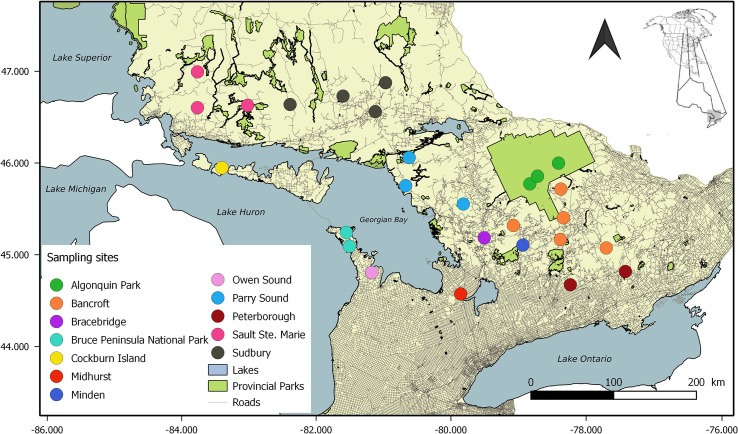
Map of southeastern Ontario sites at which American black bears (*Ursus americanus*) hair samples were collected. Bruce Peninsula sites include Bruce Peninsula National Park (light blue) and Owen Sound (light pink). Cockburn Island is indicated in yellow, and all other sites belong to the southeastern population continuum (SE-ON). Inset: North America and Ontario, with study area in grey.

In Ontario (Canada), the distribution of black bears reflects at a smaller scale what is observed across the continent: the species covers a largely intact landscape, except at the southern periphery, where fragmentation resulting from urbanization and associated infrastructures is evident (see major roads network on Fig 1). In this area, the long-term persistence of the Bruce Peninsula (BP) black bear population is uncertain. The population is indeed small (225–408 individuals [43]), and major human development and habitat fragmentation likely prevent movement to and from the larger population located in the rest of southeastern Ontario [44, 45]. From the nearest possible source areas east of Georgian Bay (Fig 1), bears would have to move 100–150 km through heavily developed areas of farmland, orchards, tourist areas (ski hills), and urban centers where there is little remaining forest cover. Analyses using both mtDNA and microsatellite loci have also shown that BP black bears are genetically differentiated from the core Ontario population, despite their close geographic proximity [14, 46]. Finally, BP black bears display reduced genetic diversity: only 2 of the 36 mitochondrial DNA haplotypes found in Ontario were identified on the BP [46], and contemporary genetic diversity is comparable to that of threatened southern populations [14].

There are several plausible reasons that could explain why BP black bears are genetically distinct. First, their low diversity could be due to a historical, postglacial colonization event (founder effect), followed by genetic drift. If this is the case, it could suggest that the population purged itself of deleterious alleles and could survive in the future with low levels of genetic diversity. Second, BP black bears could have experienced a recent demographic bottleneck because of increased mortality from large fires linked to logging and agricultural activities in the early 1900s [47]. Third, the reduced genetic diversity could have occurred when BP black bears became separated from the broader Ontario population as a result of human-induced landscape fragmentation following European settlement [14]. Finally, a combination of the aforementioned bottleneck and a sustained lack of migration due to human influences could explain the genetic differentiation of the BP population from those in the rest of the province.

We tested these alternative scenarios by comparing genetic structure and diversity measures resulting from forward simulations to those calculated from our empirical dataset. We used estimates of genetic variation from the large panmictic black bear population of southeastern Ontario to seed our model, as it was assumed to represent the undisturbed, initial genetic state of BP black bears following the Last Glacial Maximum (LGM; see [14]). We also tested the demographic bottleneck hypothesis on the BP by determining the presence of a genetic signal indicative of a recent reduction in population size [30, 31]. Finally, we used black bear mtDNA and microsatellite data from a small central Ontario island (Cockburn Island) to serve as a control representing a situation of geographic isolation with no recent bottleneck, as no sudden decrease in population size has been suspected on this island [48].

After identifying the causes of genetic isolation of BP black bears, we conducted further simulations to assess the future genetic viability of this population. Our goals were to determine if: 1) the BP population could maintain its current level of diversity under sustained isolation over the next 100 years; and 2) translocations of individuals from southeastern Ontario into the BP could help maintain or increase genetic diversity.

## Materials and methods

### Study area

The Bruce Peninsula (1,100 km^2^; 44°N 81°W) is part of the Niagara escarpment in southwestern Ontario, and separates Lake Huron from Georgian Bay (Fig 1). Although much land has been converted to agricultural, recreational, or urban use, extensive forested areas still exist. Habitat is mainly undisturbed in the north [49], especially within Bruce Peninsula National Park (BPNP– 154 km^2^ [50]). In contrast, in the southern portion of the peninsula and along the southern shores of Georgian Bay, high levels of urban development likely prevent movement of black bears between the mainland to the east and the BP [44].

### Sampling

Between 1997 and 2012, we collected black bear hair samples from live-trapped bears or from baited barbed wire hair traps [51]. In this study, we focused on sampling sites located on the BP, 9 adjacent southeastern sites (SE-ON, a subset of southeastern Ontario sampling sites), and one site located on Cockburn Island (Fig 1). All hair samples were stored dry in paper envelopes at room temperature until DNA analyses. MtDNA sequencing, individual microsatellite genotyping at 14 loci, gender determination, and estimation of genotyping error (2.18%) were performed following the conditions described in Pelletier et al. [14, 46]. Our dataset included 139 individuals from the BP and 647 individuals from SE-ON that were previously genotyped in Pelletier et al. [14], as well as 56 new individuals from Cockburn Island (the genotypes of all the individuals used in this study are provided in S1 Table). All of the Cockburn Island samples were profiled using the exact methods and quality controls as indicated in Pelletier et al. [14]. Sample collection in Bruce Peninsula National Park was approved by Parks Canada Agency. Handling procedures and sample collection on Crown land were approved annually by the Animal Care Committee of the Ontario Ministry of Natural Resources (Permits 021–97 to 021–12).

### Empirical genetic diversity and genetic structure

Of the individuals genotyped on Cockburn Island and on the BP, 51 and 81 clean mtDNA sequences were obtained, respectively. Following Pelletier et al. [46], we first performed haplotype assignment on all of these sequences, and calculated observed and standardized haplotypic diversity (h_*s*_). We also conducted a genetic structure analysis similar to the one described in Pelletier et al. [14] to determine to which Ontario genetic cluster the Cockburn Island genotypes belonged.

We calculated genetic diversity indices (allelic richness (Na); observed heterozygosity (H_O_); expected heterozygosity (H_E_)) for BP, SE-ON, and Cockburn Island samples (Fig 1), using the fastDivPart function from the R package diveRsity [52]. We assessed genetic differentiation among populations by using the same function (using 1,000 bootstrap replications) to calculate bias corrected pairwise F_ST_ [53] and Jost D [54], along with their 95% confidence intervals. Finally, we calculated inbreeding (F_IS_ [53, 55]) and average pairwise relatedness between individuals within populations [56] using 1,000 bootstrap replications, using the R package Demerelate [57].

### Forward-time simulations

We implemented 5 scenarios to model 2 populations of different sizes (N_BP_ and N_SE-ON_ representing BP and SE-ON, respectively) to identify the conditions that led to the low genetic diversity in the BP population. We extrapolated two alternative values for the initial BP population size (N_BP_ = 220 and 440) from density estimates from southern and central Ontario [44]. These extrapolated values were realistic, as they encompassed the latest population size estimates of BP black bears (N = 225–408 individuals [43]). We conducted a similar extrapolation to obtain N_SE-ON_.

**List of scenarios** (details on specific parameters and procedures used are indicated in the supplementary information in S1 File):

#### Scenario 1 –historical/high migration

Here, we modeled a scenario of high gene flow to predict what levels of genetic diversity would be expected on the BP if there had been ongoing migration between SE-ON and BP since the colonization of black bears following the LGM.

#### Scenario 2 –historical/low migration

**Fig 2:**
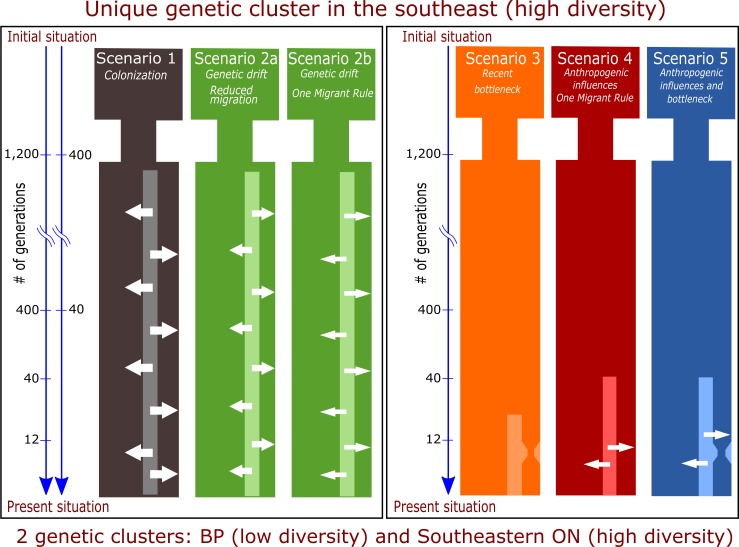
Alternative scenarios tested to understand the reasons for the genetic differentiation and reduced genetic diversity of black bears located on the Bruce Peninsula (BP). Arrow width represents various levels of migration between sites from southeastern Ontario and the BP. Two time scales are shown for scenarios 1, 2a, and 2b, as dual simulations were conducted for 1,200 and 400 generations for these scenarios.

Here, we modeled genetic variation on the BP based on little (**Scenario 2a—historical/reduced migration**) and highly reduced (**Scenario 2b—historical/one-migrant rule**) migration between SE-ON and BP following the LGM (Fig 2).

For Scenarios 1 and 2, 52 situations were modeled based on alternative migration rates, population densities, and number of generations (S1 File)

#### Scenario 3—recent bottleneck

Here, we simulated a bottleneck caused by two population crashes that were assumed to have occurred in 1903 and 1908 (S1 File), followed by population recovery. *For Scenario 3, 13 situations were modeled based on alternative mortality and growth rates (see Table A in S1 File)*.

#### Scenario 4—recently reduced migration

Here, we modeled a steady decline in migration between SE-ON and BP as a consequence of anthropogenic pressures over the last 400 years, but primarily in the past 125 years following settlement and land clearing on the peninsula. *For Scenario 4, 16 situations were modeled based on alternative migration rates and population densities (S1 File)*.

#### Scenario 5 –recent bottleneck and recently reduced migration

Here, we modeled the reduced migration induced by human activities (Scenario 4), combined with the recent population crash (Scenario 3; Fig 2). *For Scenario 5, 208 situations were modeled (13 situations of Scenario 3 combined to each of the 16 situations of Scenario 4 –S1 File)*.

**Fig 3:**
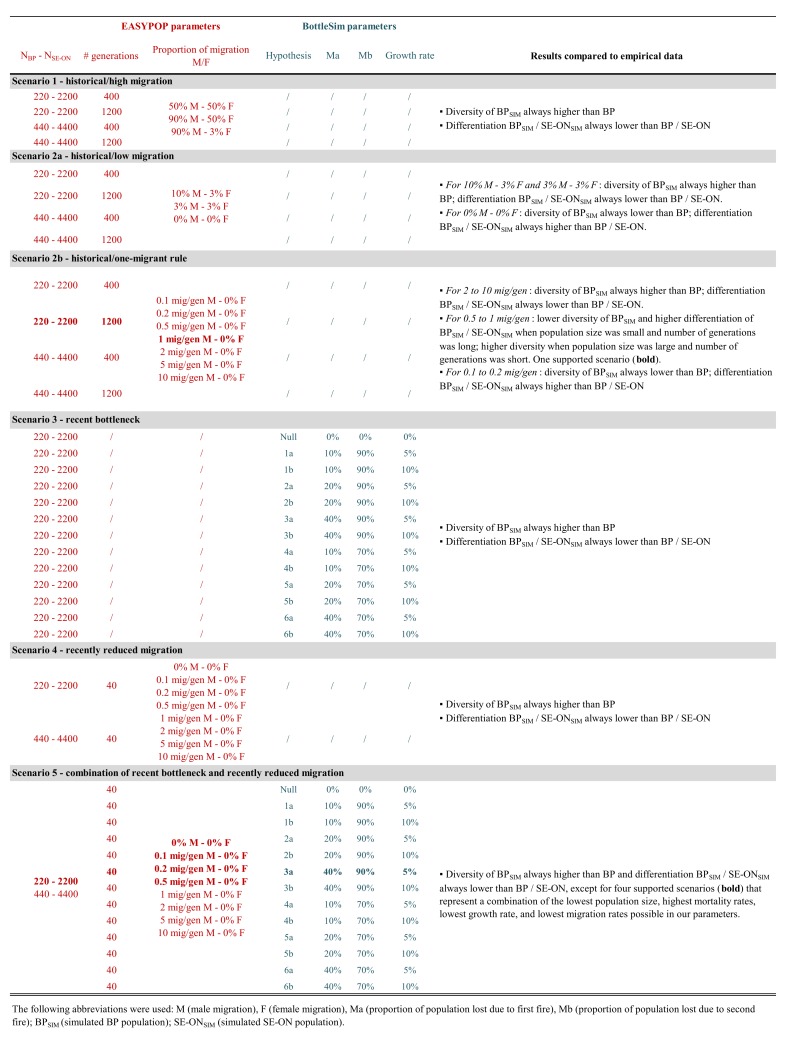
Table including all simulation parameters used for this study, and a summary of results. The following abbreviations were used: M (male migration), F (female migration), Ma (proportion of population lost due to first fire), Mb (proportion of population lost due to second fire); BP_SIM_ (simulated BP population); SE-ON_SIM_ (simulated SE-ON population).

Simulations were performed via the high-performance computer clusters offered on SHARCNET (www.sharcnet.ca). We used the program EASYPOP [22] to create the simulated populations for scenarios 1, 2 and 4 (Fig 3). We used BottleSim [23] to create the simulated populations for Scenario 3 (Fig 3). For all of our simulations (n = 289), we used 100 replicates for each parameter set (S1 File).

For each simulation output, we calculated genetic diversity and differentiation as done for the empirical data, along with the 95% confidence intervals obtained from the 100 replicates. We assessed similarity between the simulated and empirical BP populations via the overlap of the 95% confidence intervals. Scenarios were considered to explain the low genetic diversity on the BP when 2 or more simulated values from at least 2 different categories among diversity, differentiation, inbreeding, and relatedness overlapped with our empirical data.

### Effective population size

We estimated long-term effective population size (Ne_L_) for our BP samples (n = 139) via the heterozygosity-based method. As this method is highly sensitive to the mutation process, we calculated Ne_L_ under both the Infinite Allele Model (IAM, where each new mutation creates a new allele), and the Stepwise Mutation Model (SMM, where the number of alleles can stay constant, increase, or decrease at each new mutation). Under the IAM, Ne_L_ = H_E_/4μ(1—H_E_), where μ represents the mutation rate [58]. Under the SMM, Ne_L_ = [1/(1 -H_E_)^2^–1]/8μ [59].

We also estimated contemporary effective population size (Ne_C_) with a single temporal sample through: i) the linkage disequilibrium method with jacknifing implemented in LDNe 1.31 for a minimum allele frequency of 0.05 [60], and ii) the approximate Bayesian framework implemented in ONeSAMP 1.2 [61]. The estimated Ne_C_ obtained with the first method is based on the expectation that small populations accumulate more disequilibrium over time [62–64]. Under our set of circumstances (n = 139; 220 < population size < 440; number of loci = 14) the assessment of Ne_C_ is precise [65], and, as opposed to temporal methods, its value is not underestimated when samples are separated by only one generation [66, 67]. The linkage disequilibrium method is also robust to population size reductions [68], and the correction factor implemented in LDNe eliminates the bias that arises when sample size is lower than the true Ne_C_ [69, 70]. The Ne_C_ obtained with the second method relies on the estimation of the size of a Wright-Fisher population with no migration or selection based on summary statistics calculated from the empirical genotypic data. The program simulated 50,000 populations with the lower and upper limits of Ne_C_ set at 2 and 150, respectively. Priors of 10–150, 2–100, and 10–100 were also tested to assess the robustness of the results.

For comparison purposes, we calculated Ne_C_ over one black bear generation (assumed to be 10 years) through 2 temporal methods implemented in NeEstimator [71]. Individuals genotyped before 1999 were assigned to generation zero, whereas individuals identified since 2009 were assigned to generation 1. The first method, a moment based approach, estimates Ne_C_ based on changes in allele frequencies across generations [66]. The second method is a Bayesian approach based on coalescence, implemented in TM3 [72].

### Bottleneck analysis based on allelic data

We used the heterozygosity excess and the M-ratio tests to assess the presence of a bottleneck in BP black bears [29, 31]. We used the program Bottleneck [30] to compare levels of observed heterozygosity of our BP samples (H_O_) with the expected heterozygosity at mutation-drift equilibrium (H_EQ_). If there was a recent reduction in effective population size, H_O_ should be significantly larger than H_EQ_, because bottlenecks induce a faster reduction in allelic diversity than heterozygosity. To incorporate uncertainties in the mutation process [73], we used both a stepwise mutation model (SMM), and a two-phase model (TPM [74]) that involved 10% and 30% of multi-step mutations (*p*_g_) (see [30, 73]). We used both 3.1 and 3.5 as the mean size of multi-step mutations (*δ*_g_). We ran the analysis for 10,000 replications, and assessed bottleneck significance via the Wilcoxon signed rank test [30].

The M-ratio test supposes that bottlenecked populations experience a faster reduction in the number of alleles (k) than in the range of allele size (r; measured in repeat units) due to a loss of rare alleles from genetic drift. The M-ratio is then calculated as M = k/r. For a bottleneck to be identified, M should fall under a critical value, Mc (calculated via Critical_M), in 95% of the cases or above. The M-ratio is generally expected to be lower than 0.7 for bottlenecked populations, and higher than 0.8 for the others [31]. To obtain Mc, we calculated the pre-bottleneck θ parameter (θ = 4Neμ), with a mutation rate of μ = 2*10^−4^. We used the N_BP_ and Ne_C_ values from the linkage disequilibrium and temporal methods to seed our M-ratio test. N_BP_ represented a realistic upper bound of the effective population size (Ne), as it incorporates individuals of any age and sex category, whereas the Ne statistics only includes reproductive individuals. We used 10,000 replications, with *δ*_*g*_ = 3.1 and 3.5 and *p*_*g*_ = 0.1 and 0.2, as suggested in Piry et al. [30] and Peery et al. [73].

### Future of the BP population–effect of translocations

We used the R package AlleleRetain [75] to determine if regular translocations of SE-ON individuals into the BP could increase allele retention and prevent inbreeding over the next 10 generations. Based on our EASYPOP parameters, we ran simulations with population sizes set at n = 2,200 and 220, or n = 4,400 and 440 individuals for the source (N_SE-ON_) and start (N_BP_) populations, respectively. The goal was to determine if the current BP population could maintain its genetic diversity over time under sustained isolation from SE-ON.

For all simulations, we considered rare alleles to be those present in 5% or less of the source population. Other simulation parameters were based on information on the demography of Ontario black bears [76, 77], and included: polygynous/seasonal mating system; 0.5 sex ratio; reproductive age from 4 to 20 years old (with senescence at 16 years old); reproductive output influenced by age; annual adult survival rate of 0.9. We ran these simulations over 100 years by including translocations of 0, 5 or 10 bears from the source population every 5 or 10 years, and used 1,000 replications for each parameter set. To fully assess the effects of translocations on allele retention and inbreeding, all translocated individuals were given breeding opportunities.

## Results

### Empirical genetic diversity and genetic structure

The genetic differentiation levels observed between BP and SE-ON and between Cockburn Island and SE-ON were equivalent (see overlap of 95% CIs of Jost D and F_ST_ in Table 1). However, unlike BP black bears, Cockburn Island individuals did not represent a single genetic cluster of their own; rather, they were part of the southeastern Ontario cluster identified in Pelletier et al. [14], and displayed a high cluster membership value (*q* = 0.9).

**Table 1:** Genetic differentiation measures obtained from empirical data.

	SE-ON	Cockburn Island	BP
SE-ON	**-**	*0*.*102 [0*.*090–0*.*114]*	*0*.*122 [0*.*114–0*.*129]*
Cockburn Island	**0.265 [0.242–0.289]**	**-**	*0*.*191 [0*.*175–0*.*209]*
BP	**0.282 [0.266–0.297]**	**0.208 [0.181–0.237]**	**-**

Bold: Jost-D [95% CI]; italics: F_ST_ [95% CI].

BP black bears had a lower allelic richness and a lower observed and expected heterozygosity than Cockburn Island individuals (Table 2). Two mtDNA haplotypes were identified on the BP (n = 81; HAP2 and 5; GenBank accession numbers GU724159 and GU724161) against 4 on Cockburn Island (n = 51; HAP1, HAP5, HAP24, and HAP25; GenBank accession numbers GU724158, GU724161, GU724179, and GU724180 [46]). Standardized haplotypic diversity (h_*s*_) was also higher on Cockburn Island than on the BP (h_*s*_ = 3.8 and 2, respectively).

**Table 2:** Diversity and differentiation statistics of empirical data and supported simulations.

		EASYPOP parameters		BottleSim parameters			Results: diversity statistics			Results: differentiation statistics		Results: inbreeding statistics		Results: relatedness
Data	n	# generations	Proportion of migration M/F	Ma	Mb	Growth rate	Allelic richness	Ho	H_E_	F_ST_	Jost D	F_IS_- Nei	F_IS_- Weir	*r*
*Empirical*														
SE-ON	647	/	/	/	/	/	10.53	0.77	0.81			0.05	0.05	-0.005
Cockburn Island	56	/	/	/	/	/	5.23	0.65	0.7	0.102	0.265	-0.036	-0.036	0.267
BP	139	/	/	/	/	/	4.28	0.55	0.55	0.122	0.282	0.022	0.017	0.44
*Simulations*														
Scenario 2b	220–2200	1200	1 mig/gen M—0% F	/	/	/	4.65 [4.59–4.71]	**0.55 [0.54–0.57]**	**0.56 [0.54–0.57]**	0.156 [0.152–0.160]	0.222 [0.216–0.227]	0.006 [0.000–0.012]	0.006 [0.000–0.012]	**0.444 [0.433–0.455]**
Scenario 5	220–2200	40	0% M—0% F	40%	90%	5%	**4.22 [4.15–4.28]**	0.60 [0.60–0.61]	0.60 [0.59–0.61]	**0.123 [0.118–0.127]**	0.248 [0.237–0.258]	0.001 [-0.006–0.008]	0.001 [-0.006–0.008]	0.400 [0.392–0.408]
Scenario 5	220–2200	40	0.1 mig/gen M—0% F	40%	90%	5%	**4.28 [4.21–4.35]**	0.61 [0.60–0.62]	0.60 [0.60–0.61]	**0.120 [0.116–0.124]**	0.246 [0.237–0.255]	0.000 [-0.008–0.007]	-0.001 [-0.008–0.007]	0.394 [0.386–0.402]
Scenario 5	220–2200	40	0.2 mig/gen M—0% F	40%	90%	5%	**4.31 [4.24–4.37]**	0.61 [0.60–0.62]	0.60 [0.60–0.61]	**0.120 [0.116–0.125]**	0.245 [0.235–0.255]	-0.004 [-0.010–0.002]	-0.004 [-0.010–0.002]	0.396 [0.387–0.404]
Scenario 5	220–2200	40	0.5 mig/gen M—0% F	40%	90%	5%	**4.34 [4.27–4.40]**	0.62 [0.61–0.62]	0.61 [0.60–0.62]	**0.119 [0.114–0.124]**	0.242 [0.232–0.252]	-0.002 [-0.007–0.003]	-0.002 [-0.007–0.003]	0.388 [0.380–0.396]

Statistics shown: allelic richness; observed heterozygosity (H_O_); expected heterozygosity (H_E_); F_ST_; Jost D; F_IS_-Nei; F_IS_-Weir; *r* (relatedness). 95% CIs (over 100 replicates) are included for all statistics calculated from the simulated data. Overlapping values with the empirical data are indicated in bold. Two or more values from at least 2 different categories among diversity, differentiation, inbreeding and relatedness had to overlap for a simulation to be considered as supported. The following abbreviations were used: n (number of individuals), M (male migration), F (female migration), Ma (proportion of population lost due to first fire), Mb (proportion of population lost due to second fire).

### Forward-time simulations

For Scenarios 1 (historical—high migration) and 2a (historical—reduced migration), all simulated situations showed higher levels of diversity and lower differentiation than our empirical data, except in the absence of migration, for which lower levels of diversity and higher differentiation were detected compared to what was observed on the BP (Fig 3).

Under Scenario 2b (historical—one-migrant rule), simulations that included 0.1 to 0.2 migrants per generation provided lower levels of diversity than on the BP (Fig 3). Simulations with 2 or more migrants per generation always showed higher levels of diversity and lower differentiation than on the BP (Fig 3). At 0.5 and 1 migrants per generation, results varied depending on population size and number of generations. Only one of these simulations led to results corresponding to the BP situation (1 migrant per generation; N_BP_ = 220; N_SE-ON_ = 2,200; number of generations = 1,200 –Table 2 and Fig 3).

Results from Scenarios 3 (recent bottleneck) and 4 (recently reduced migration) showed higher levels of diversity than our empirical BP data in all situations, rejecting these hypotheses as the sole factor causing low diversity in BP black bears (Fig 3).

The supported scenario 5 simulations (combination of Scenarios 3 and 4) corresponded to a range of 0 to 0.5 migrants per generation over 40 generations, N_BP_ = 220, and N_SE-ON_ = 2,200. The associated bottleneck included a 40% initial mortality drop, followed by 90% mortality, and a 5% growth rate (BottleSim hypothesis 3a –Table 2 and Fig 3). For this scenario, other simulations approached BP’s empirical results based on allelic richness and genetic differentiation, however, the observed and expected heterozygosity were always higher than the empirical data (Table 2 and Fig 3).

Results for all simulations (supported and unsupported) are provided in S2 Table.

### Effective population size

Based on a mutation rate of 2*10^−4^, Ne_L_ for the BP population ranged from 1,527 under the IAM, to 2,461 under the SMM. Ne_C-LD_ was estimated at 15.4 (95% CI = [12.6–18.5]—Table 3), which was lower than all of the estimates from the Bayesian method implemented in ONeSAMP. Generally, ONeSAMP estimations were not sensitive to the choice of priors, as the only results that did not overlap were for priors 2–150 (Ne_C-2-150_ = 25.6, 95% CI = [21.4–35.2]) and 10–150 (Ne_C-10-150_ = 42.6, 95% CI = [36.8–56.4]).

**Table 3:** Estimation of contemporary effective population size (Ne) of BP and the associated 95% CIs.

	Mean Ne	95% CI
**Single sample**		
*Bayesian—ONeSAMP*		
Priors 2–150 –Ne_C-2-150_	25.6	[21.4–35.2]
Priors 2–100 –Ne_C-2-100_	33.8	[28.1–46.6]
Priors 10–150 –Ne_C-10-150_	42.6	[36.8–58.4]
Priors 10–100 –Ne_C-10-100_	37.9	[33.2–47.9]
*Linkage disequilibrium—LDNe-* Ne_C-LD_		
BP—n = 139	15.4	[12.6–18.5]
n = 20	21.1	[11.4–54.6]
n = 50	16.8	[12.5–22.6]
n = 100	18	[14.5–22.3]
SE-ON—n = 647	560.8	[461.9–698.1]
**Temporal—NeEstimator**		
*Moment based temporal–*Ne_C-Moment_	56.2	[44.6–69.3]
*Bayesian–coalescence TM3 –*Ne_C-Coal_	22.8	[22.5–23]

Analyses were conducted with both single sample (LDNe, ONeSAMP), and temporal methods (NeEstimator). Analyses based on the linkage disequilibrium method with jacknifing include various sample sizes (n) for BP, and empirical SE-ON data. ONeSAMP analyses included various priors for upper and lower Ne.

The moment based approach based on 2 temporal samples resulted in a Ne_C_ similar to Ne_C-10-150_ (Ne_C-Moment_ = 56.2; 95% CI = [44.6–69.3]), whereas the estimates from the Bayesian coalescence approach (Ne_C-Coal_ = 22.8; 95% CI = [22.5–23.0]), were similar to Ne_C-2-150_ (Table 3).

### Recent bottleneck (allelic data)

Bottleneck analyses for BP black bears did not detect the presence of a heterozygosity excess under the SMM nor the TPM (Table 4). Allele frequency distributions did not illustrate the presence of a mode shift, which can illustrate a bottleneck. In contrast, our M-ratio test detected a bottleneck in BP black bears. The observed M (0.699) was always lower than Mc (Table 5), which is indicative of a recently reduced population size.

**Table 4:** Results of bottleneck heterozygosity excess tests based on allelic data for our BP samples.

	Expected number of loci with heterozygosity excess	# loci with heterozygosity deficiency vs. excess	Probability excess (Wilcoxon test)	Mode shift
*δ*_g_ = 3.1–*σ*^2^_g_ = 12				
TPM-70%	8.22	5:9	0.097	no
TPM-90%	8.25	6:8	0.548	no
SMM	8.28	6:8	0.821	no
*δ*_g_ = 3.5–*σ*^2^_g_ = 16				no
TPM-70%	8.2	5:9	0.086	no
TPM-90%	8.24	6:8	0.524	no
SMM	8.28	6:8	0.821	no

Three mutation models were used (the TPM two-phase model from 70% to 90% of single step mutations, and the SMM stepwise mutation model). The probability to detect a bottleneck was assessed with the Wilcoxon one-tailed test. P-values less than 0.05 are indicated in bold. The presence or absence of a mode-shift is also indicated. For comparison purposes, 2 sets of parameters were used for *δ*_g_ (the mean size of multi-step mutations) and *σ*^2^_g_ (the variance among multi-step mutations) following the recommendation of Piry et al. [30] and Peery et al. [73].

**Table 5:** Results of M-ratio tests.

Ne	# loci	θ	*δ*_*g*_	*p*_*g*_	Mc	P
*Estimations based on bear densities* (Ne = N_BP_)						
220	14	0.176	3.1	0.1	0.866	0.0000
220	14	0.176	3.1	0.2	0.798	0.0012
220	14	0.176	3.5	0.1	0.855	0.0000
220	14	0.176	3.5	0.2	0.777	0.0024
440	14	0.352	3.1	0.1	0.855	0.0000
440	14	0.352	3.1	0.2	0.783	0.0026
440	14	0.352	3.5	0.1	0.839	0.0001
440	14	0.352	3.5	0.2	0.755	0.0058
*Estimations based on genetic estimates* (Ne = Ne_C_)						
15.4	14	0.012	3.1	0.1	0.881	0.0000
15.4	14	0.012	3.1	0.2	0.814	0.0005
15.4	14	0.012	3.5	0.1	0.869	0.0000
15.4	14	0.012	3.5	0.2	0.799	0.0006
22.8	14	0.018	3.1	0.1	0.879	0.0000
22.8	14	0.018	3.1	0.2	0.814	0.0002
22.8	14	0.018	3.5	0.1	0.869	0.0000
22.8	14	0.018	3.5	0.2	0.799	0.0016
56.2	14	0.045	3.1	0.1	0.878	0.0000
56.2	14	0.045	3.1	0.2	0.807	0.0002
56.2	14	0.045	3.5	0.1	0.865	0.0000
56.2	14	0.045	3.5	0.2	0.795	0.0010

Tests based on effective population sizes calculated from: i) demographic data (N_BP_ = 220, 440), and ii) genetic data based on single and multiple temporal samples (Ne_C_ = 15.4, 22.8, and 56.2), with a mutation rate of μ = 2x10^-4^. Mc represents the value above which 95% of the M-ratio values (M) should be found. M is calculated as the number of alleles divided by the range in allele size. P represents the proportion of replicates found below the observed M-ratio. The M-ratio for our BP dataset (averaged over 14 loci) was M = 0.699.

### Future of the BP population–effect of translocations

**Fig 4:**
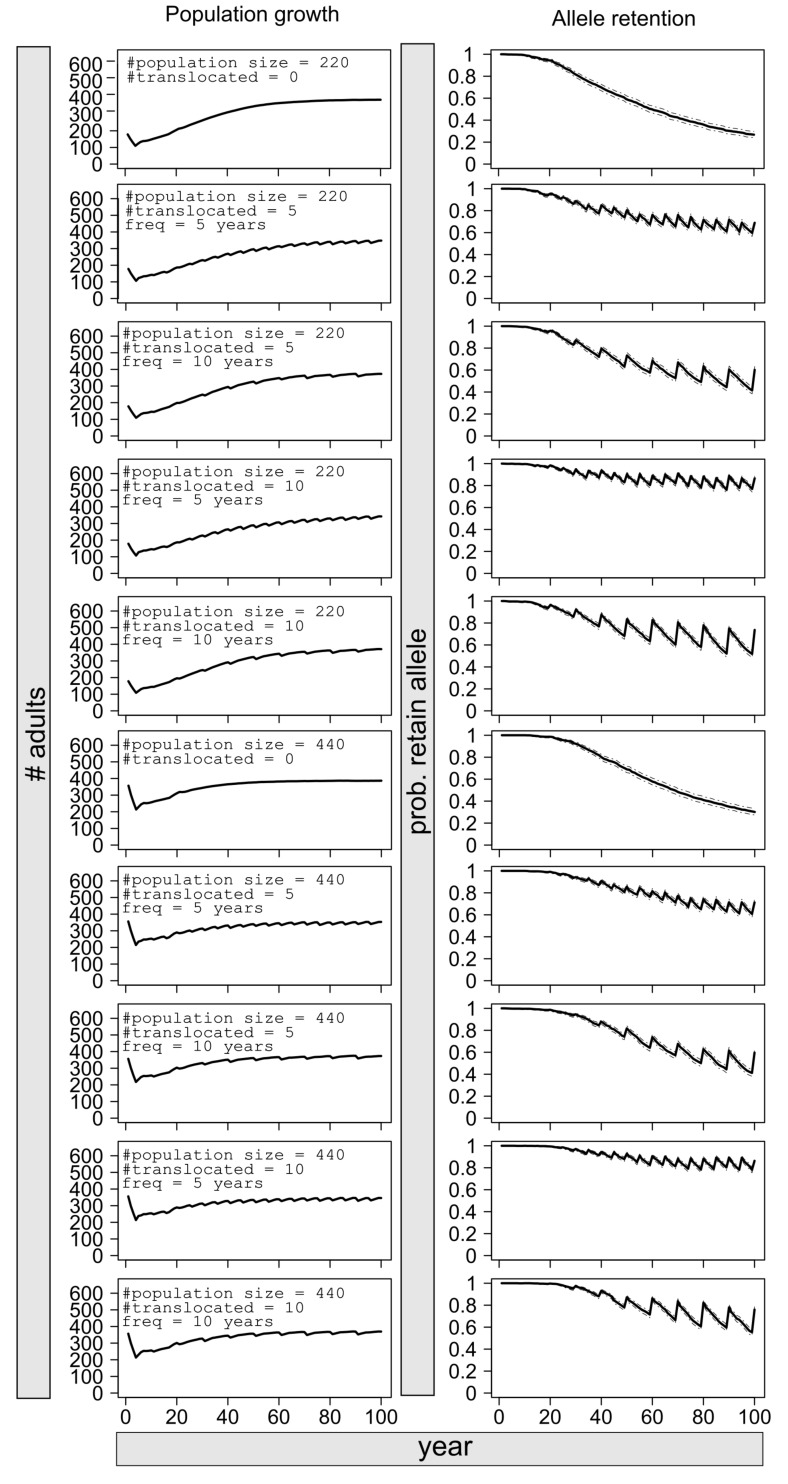
Population growth and allele retention of the BP population over a 100-year period, under varying translocation scenarios (0, 5 or 10 bears successfully translocated; translocations every 5 or 10 years). Simulations were conducted in AlleleRetain [75].

Depending on starting population size (N_BP_ = 440 or 220), BP black bears could lose 70 to 80% of their rare alleles over the next 100 years under sustained isolation (Fig 4). Successfully translocating 5 to 10 bears from SE-ON into the BP every 10 years would slow down the loss of allelic diversity, but the population could still lose 30 to 50% of these alleles during that period (Fig 4). Allele retention would be more efficient with translocations of 5 to 10 bears every 5 years, as the reduction in allelic diversity would reach maximum 30% (Fig 4).

## Discussion

In Ontario, American black bears are continuously distributed and show a clinal genetic structure due to isolation-by-distance, except for the BP population, which is genetically differentiated from the core and displays reduced diversity [14]. Our simulations support the hypothesis that this pattern is the consequence of a recent demographic bottleneck combined with reduced gene flow, rather than of reduced gene flow following the LGM. These results illustrate that local populations of large carnivores can differentiate themselves quickly from the core when under pressure from landscape fragmentation and demographic conditions inducing small population size. This is important to note, as human development in areas that are currently free of ecological disturbances may lead to rapid habitat loss and fragmentation in those very species [16, 17].

Conservation concerns regarding the BP population have already been raised by previous demographic and ecological analyses, which suggest that if this population was further restricted geographically, removing sources of non-natural mortality would not be enough to ensure its persistence [44, 45]. Though these are the most urgent matters regarding BP black bears viability, our analyses also show that without intervention, this population would lose further genetic diversity.

American black bears are considered an ecological indicator on the Bruce Peninsula, [78], and as such, it is important to preserve the BP population despite its absence of unique alleles. Here, we tested for alternative translocation scenarios to find ones that would enhance retention of alleles brought in by immigrants. These translocation scenarios could be combined with previous management recommendations so that this population can persist without losing further genetic variation.

### Reasons for the low diversity on the BP

As shown by our rejection of Scenarios 1 and 2a, the BP population could not have reached its current level of diversity through genetic drift if historical migration was high. One situation of highly reduced historical gene flow was supported by our simulations (Scenario 2b; 1 migrant per generation over 1,200 generations); however, considering the dispersal abilities of black bears (young males can travel distances up to 200 km or more [79, 80]), it is unlikely that their migration rate was this low for this long, especially following the return of forests in the Great Lakes region 4,000 years ago [81].

In addition to reduced gene flow, rapid declines in population size decrease genetic diversity, which may hinder population viability (e.g., greater prairie chickens [82]; Florida panthers [9]). The reduced diversity of BP black bears [14, 46] is unlikely to have been solely caused by a recent bottleneck, as none of the simulations from Scenario 3 were supported. However, a range of simulated data from Scenario 5 (combination of recently reduced migration and bottleneck) overlapped with BP diversity measures, suggesting that recently reduced migration associated with 2 sudden drops in population size 5 years apart could explain the observed pattern.

The presence of a bottleneck in BP black bears was also supported by the M-ratio test, although this signal was not detected by heterozygosity excess tests. Thus, among the alternative scenarios that were supported by our simulations, a combination of a demographic bottleneck and recently reduced migration (Scenario 5) seems the most plausible to explain the reduced diversity of the BP population.

There are several explanations as to why we obtained contrasting results from the M-ratio and heterozygosity excess tests. First, the heterozygosity excess test could have suffered from a lack of statistical power [83]. Second, a false positive may have occurred in the M-ratio test if our original assumption that BP and SE-ON initially formed a panmictic unit was wrong [84], although this is unlikely considering the isolation-by-distance structuring pattern of black bears across Ontario [14]. Third, the M-ratio test has been shown to perform better than the heterozygosity excess test in particular situations [85, 86]. As an example, the M-ratio test is able to detect severe bottlenecks that are followed by demographic recovery, whereas the heterozygosity excess test does better on recent and less severe bottlenecks [29, 73, 87]. Finally, it is plausible that these contrasting results illustrate a higher sensitivity of allelic richness than of heterozygosity to bottlenecks. In this situation, recently bottlenecked populations can show high heterozygosity while displaying low allelic richness [88]. We found this pattern in our supported Scenario 5 simulations, for which observed and expected heterozygosity were always higher than our empirical data, whereas allelic richness and F_ST_ overlapped. These inherent differences in how each statistic responds to specific demographic processes likely explain why our supported scenarios only matched with two or three of the eight indices used to assess fit to the empirical data (see S2 Table).

Despite the possible limitations of bottleneck tests and simulations, which can only approach natural situations, two elements support the hypothesis that low migration alone did not lead to the reduced genetic variability observed on the BP. First, BP black bears had a lower genetic diversity than individuals located on Cockburn Island. Second, Cockburn Island individuals grouped with the SE-ON cluster and displayed a high cluster membership value combined with a low level of admixture. Both of these factors suggest that a low level of migration exists from the mainland and neighboring islands onto Cockburn Island. On this island however, reduced migration was not enough to result in a genetic diversity level as low as that observed for BP black bears. This indicates that additional factors that induce low genetic variation are at play on the BP. Examples of these include population size reduction on the BP, as well as a smaller gene pool in individuals migrating to BP compared to ones migrating to Cockburn Island.

Additional genetic data such as allelic richness, heterozygosity, and haplotypic diversity obtained from black bears inhabiting islands located in the vicinity of the BP, combined with powerful models simulating the colonization and demographic history of the species in Ontario, could help confirm the impact of recent bottlenecks on current genetic diversity. Further, landscape genetic simulations could be conducted to identify areas where spatial bottlenecks that may be detrimental to genetic diversity could occur [89].

### Future of the BP population–effect of translocations

All our estimates of effective population size (mean Ne_C_ = 15.4 to 56.2) were lower than expected considering the estimated black bear abundance on the BP (N_BP_ = 225–408 [43]). The variation in Ne_C_ could be due to a violation of one or more of the assumptions of constant population size, closed population, and overlapping generations, or to differences in the underlying models used by each software. Relatedness among BP individuals is also high [14], and even at carrying capacity, the size of the population will remain small due to the reduced area of bear habitat in the region [44, 45]. These elements are important to note, as detrimental phenotypic and physiologic consequences of small population size and reduced genetic variation may already be underway but going unnoticed on the BP.

Based on the relationship between inbreeding coefficient and effective population size, we calculated that inbreeding could increase from 1 to 3% per generation in BP black bears. The deleterious effects of inbreeding on population persistence [1] have led to the implementation of genetic rescue efforts in several species [90, 91]. For Florida black bears [8], negative manifestations of inbreeding such as cryptorchidism have been reported [92], and genetic variation is the lowest observed in any black bear population (H_E_ = 0.27 [8]). In Florida panthers, phenotypic indications of inbreeding were detected when observed heterozygosity was at 0.101 [9]. Although heterozygosity levels of BP black bears are not as low as described above, our simulations show that sustained immigration or regular translocations of individuals from SE-ON onto the BP are necessary to prevent further loss of allelic diversity. Indeed, 10 generations with no gene flow between SE-ON and BP would be sufficient for the population to lose 70 to 80% of its rare alleles.

When only taking distance into account, the SE-ON and BP populations could be considered as having the ability to reconnect. Indeed, several records of black bears travelling over much longer distances than 100–150 km exist [80, 93, 94]. The main barrier to this process is the highly developed and fragmented landscape south of Georgian Bay (Fig 1). Translocations of black bears have been successful over longer distances than 150 km, but homing behavior becomes an issue when distances between the capture and release site are short [95]. In a study conducted in Arkansas, over 10% of translocated bears attempted to return to their capture site, which was only 160 km away from the release site [96]. The immediate concern with homing behavior is road mortality, which can account for over 50% of mortality the year following release [97].

Our results show that successfully translocating 5 to 10 bears every 5 years would help to retain allelic diversity. However, survival of translocated bears can range from 0.2 to 0.624 the year post-release [96–98]. Based on this information, the number of SE-ON individuals needing to be translocated on the BP every 5 years could range from 9 to17 if survival was as high as 0.624, and from 25 to 50 if survival was as low as 0.2. In our simulations, we also assumed that all translocated bears had breeding opportunities. In reality, migrants may not always be able to breed [99]. Practically, this represents a limitation for translocation programs that aim to enhance genetic diversity. In black bears however, even a small founder group can form a genetically diverse reintroduced population, and stay genetically sustainable for at least a decade [100]. This suggests that reintroduced individuals do find enough breeding opportunities for a translocation program to be successful.

Our genetic results further suggest that allowing for the possibility of a continuous intake of migrants spread over several years would enable the population to better retain diversity than if the population was to remain isolated and received translocated individuals. Indeed, under isolation, the diversity brought in by the new migrants could be lost after a short period of time (Fig 4). Considering that it is unlikely that connectivity between BP and SE-ON will be reestablished, an initial large translocation effort of sexually mature individuals from SE-ON could be used to boost genetic diversity. Subsequent smaller translocations would then help to retain new alleles and maintain or increase genetic diversity.

To carry out a successful translocation program, it will be critical to select conditions that will maximize the survival probability and breeding opportunities of translocated bears. These conditions integrate choice of habitat, individuals to translocate, capture and release technique, public acceptance of a translocation program, and follow-up monitoring. As such, we recommend:

- **For translocation logistics**:
  - Because causes of human–bear conflict are controversial [101], consulting extensively with local stakeholders to secure acceptance of a translocation program prior to initiating any translocations.
  - Favoring soft winter release over summer release, as survival of translocated individuals is significantly higher for soft winter releases (0.88 vs. 0.2 [98]).
  - Conducting a larger translocation event followed by subsequent smaller translocations to boost and subsequently retain genetic diversity.
  - Favoring the frequency of translocations over the number of individuals translocated.
  - Avoiding selecting the lowest possible number of individuals to translocate in the event that these individuals would have difficulty surviving and reproducing.
- **For habitat and landscape management:**
  - Ensuring that bears are released in good quality habitat, as food shortages have been suggested to be associated with long distance movements [94].
  - Protecting bear habitat on the BP, particularly outside of the National Park [44].
- **For the choice of individuals:**
  - Choosing individuals that are less likely to travel back to their capture site, such as subadults and encumbered females [95].
  - Avoiding the release of food conditioned individuals [102, 103].
- **For follow-up management:**
  - Lowering harvest rates the first year post-release to allow enough time for individuals to establish themselves and reach a high survival rate, which can be as high as 0.9 the 2^nd^ year post-release [96].
  - Conducting other programs aiming at reducing sources of non-natural mortality.

## Conclusion

Our results provide additional information to the demographic data that suggested that BP black bears were of conservation concern [44]. Here, we show that a risk of inbreeding exists based on relatedness and population size estimates, and that genetic diversity is unlikely to be maintained under sustained isolation (Fig 4).

In North America, anthropogenic activities inducing habitat loss and fragmentation are increasing, and moving northward [16, 17, 104]. This, with the addition of climate change influencing future resource availability, suggests that caution should be taken when implementing land use plans, as black bears located in portions of the range such as central Ontario could become more geographically isolated. Such isolation could increase the probability of demographic bottlenecks, and, in the event of landscape fragmentation, these isolated populations may experience higher mortality rates as a result of human–bear conflict and vehicular traffic. As such, we suggest that the future of BP black bears, based on both the genetic and ecological context, is fully linked with landscape management.

Translocation efforts to boost genetic diversity have been successful in Greater prairie chickens [105] and Florida panthers [90]. For BP black bears, the efficiency of translocations would be increased if they were coupled with landscape management aimed to conserve or create natural corridors between BP and SE-ON. This method would allow sustained black bear migration into the BP, hereby enhancing genetic diversity and retaining evolutionary potential in the long-term. However, the economic and ecological contexts in areas south and east of Georgian Bay are unlikely to allow for landscape management actions that re-create migration corridors. Thus, regularly supplementing the BP population with SE-ON individuals following a large translocation effort could be a viable alternative to boost genetic diversity. These translocations would have a dual effect, as they would also be helpful to keep the population size at a level that would ensure long-term persistence.

If public acceptance is obtained, the proposed translocation program will require a well thought-out choice of capture technique, individuals to translocate, and release site, as well as a close monitoring of the translocated bears to evaluate success and avoid potential human-bear conflicts stemming from future translocations [101, 106].

Overall, our study enabled us to estimate the migration rates and timeframes necessary for populations to go from high to intermediate levels of diversity. Our results also enabled us to make recommendations to enhance the genetic diversity of BP black bears. Our approach can be applied to other vagile species to determine at which point concerns regarding the persistence of isolated populations should be raised, and thus inform long-term and large-scale management plans for populations that are becoming increasingly small and fragmented.

## Supporting information

S1 File Justifications of parameters and procedures used in forward-time simulations. Simulations were performed in EASYPOP (Balloux 2001) and in BottleSim (Kuo and Janzen 2003), via the high-performance computer clusters offered on SHARCNET (www.sharcnet.ca). Results for all simulations are provided in S2 Table.
(DOCX)

S1 Table Genotypes of black bears used in this study. This file contains the multilocus genotypes for the 842 American black bears used in this study. The data are formatted with two columns per locus.
(XLSX)

S2 Table Simulation results. This file contains the results of all of the simulations conducted in this study, and provides the parameters used in EASYPOP (Balloux 2001). Results include genetic diversity, differentiation and inbreeding statistics, as well as a relatedness statistic. Supported simulations are indicated in bold, and alternative scenarios are color coded.
(XLSX)

## References

[1] Frankham R. Inbreeding and extinction: a threshold effect. Conserv Biol. 1995;9:792–799.

[2] Frankham R. Do Island populations have less genetic variation than mainland populations? Heredity. 1997;78:311–327. 9119706 10.1038/hdy.1997.46

[3] Lande R. Risks of population extinction from demographic and environmental stochasticity and random catastrophes. Am Nat. 1993;142:911–927. 29519140 10.1086/285580

[4] Keyghobadi N. The genetic implications of habitat fragmentation for animals. Can J Zool. 2007;85:1049–1064.

[5] Cegelski CC, Waits LP, Anderson NJ. Assessing population structure and gene flow in Montana wolverines (Gulo gulo) using assignment-based approaches. Mol Ecol. 2003;12:2907–2918. 14629372 10.1046/j.1365-294x.2003.01969.x

[6] McRae BH, Beier P, Dewald LE, Huynh LY, Keim P. Habitat barriers limit gene flow and illuminate historical events in a wide-ranging carnivore, the American puma. Mol Ecol. 2005;14:1965–1977. 10.1111/j.1365-294x.2005.02571.x 15910319

[7] Proctor MF, McLellan BN, Strobeck C, Barclay RM. Genetic analysis reveals demographic fragmentation of grizzly bears yielding vulnerably small populations. Proceedings of the Royal Society of London B: Biological Sciences. 2005;272(1579):2409–2416. 10.1098/rspb.2005.3246 PMC1559960 16243699

[8] Dixon JD, Oli MK, Wooten MC, Eason TH, McCown JW, Cunningham MW. Genetic consequences of habitat fragmentation and loss: the case of the Florida black bear (Ursus americanus floridanus). Conserv Genet. 2007;8:455–464.

[9] Culver M, Hedrick PW, Murphy K, O’Brien S, Hornocker MG. Estimation of the bottleneck size in Florida panthers. Animal Conserv. 2008;11:104–110.

[10] Laliberté AS, Ripple WJ. Range contractions of North American carnivores and ungulates. BioScience. 2004;54:123–138.

[11] Wiegand T, Revilla E, Moloney KA. Effects of habitat loss and fragmentation on population dynamics. Conserv Biol. 2005;19:108–121.

[12] Zigouris J, Dawson FN, Bowman J, Gillett RM, Schaefer JA, Kyle CJ. Genetic isolation of wolverine (Gulo gulo) populations at the eastern periphery of their North American distribution. Conserv Genet. 2012;13:1543–1559.

[13] Sacks BN, Brown SK, Ernest HB. Population structure of California coyotes corresponds to habitat-specific breaks and illuminates species history. Mol Ecol. 2004;13:1265–1275. 10.1111/j.1365-294X.2004.02110.x 15078462

[14] Pelletier A, Obbard ME, Mills K, Howe EJ, Burrows FG, White BN, et al Delineating genetic groupings in continuously distributed species across largely homogeneous landscapes: a study of American black bears (Ursus americanus) in Ontario, Canada. Can J Zool. 2012;90:999–1014.

[15] Stronen AV, Forbes GJ, Paquet PC, Goulet G, Sallows T, Musiani M. Dispersal in a plain landscape: short-distance genetic differentiation in southwestern Manitoba wolves, Canada. Conservation Genetics. 2012;13(2):359–371.

[16] Cardillo M, Mace GM, Gittleman JL, Purvis A. Latent extinction risk and the future battlegrounds of mammal conservation. Proc Natl Acad Sci USA. 2006;103:4157–4161. 10.1073/pnas.0510541103 16537501 PMC1449663

[17] Cardillo M, Mace GM, Gittleman JL, Jones KE, Bielby J, Purvis A. The predictability of extinction: biological and external correlates of decline in mammals. Proc R Soc London B. 2008;275:1441–1448. 10.1098/rspb.2008.0179 PMC2602711 18367443

[18] Paetkau D, Shields GF, Strobeck C. Gene flow between insular, coastal and interior populations of brown bears in Alaska. Mol Ecol. 1998b;7:1283–1292. 9787441 10.1046/j.1365-294x.1998.00440.x

[19] Walker CW, Vila C, Landa A, Linden M, Ellegren H. Genetic variation and population structure in Scandinavian wolverine (Gulo gulo) populations. Mol Ecol. 2001;10: 53–63. 11251787 10.1046/j.1365-294x.2001.01184.x

[20] Milot E, Weimerskirch H, Duchesne P, Bernatchez L. Surviving with low genetic diversity: the case of albatrosses. Proc R Soc London B. 2007;274(1611):779–787. 10.1098/rspb.2006.0221 PMC2093973 17251114

[21] Johnson JA, Tingay RE, Culver M, Hailer F, Clarke ML, Mindell DP. Long‐term survival despite low genetic diversity in the critically endangered Madagascar fish‐eagle. Mol Ecol. 2009;18(1):54–63. 10.1111/j.1365-294X.2008.04012.x 19140964

[22] Balloux F. EASYPOP (Version 17): A computer program for population genetics simulations. J Hered. 2001;92:301–302. 11447253 10.1093/jhered/92.3.301

[23] Kuo C, Janzen F. BottleSim: a bottleneck simulation program for long-lived species with overlapping generations. Mol Ecol Notes. 2003;3:669–673.

[24] Currat M, Ray N, Excoffier L. SPLATCHE: a program to simulate genetic diversity taking into account environmental heterogeneity. Mol Ecol Notes 2004;4:139–142.

[25] Cornuet JM, Santos F, Beaumont MA, Robert CP, Marin JM, Balding DJ, et al Inferring population history with DIYABC: a user-friendly approach to Approximate Bayesian Computations. Bioinformatics. 2008;24:2713–2719. 10.1093/bioinformatics/btn514 18842597 PMC2639274

[26] Hoban S, Bertorelle G, Gaggiotti OE. Computer simulations: tools for population and evolutionary genetics. Nat Reviews Genet. 2012;13:110–22. 10.1038/nrg3130 22230817

[27] Carvajal-Rodriguez A. Simulation of genomes: a review. Curr Genomics. 2008;9:155–159. 10.2174/138920208784340759 19440512 PMC2679650

[28] Arenas M. Simulation of molecular data under diverse evolutionary scenarios. PLoS Computational Biol. 2012;8:e1002495. 10.1371/journal.pcbi.1002495 PMC3364941 22693434

[29] Cornuet JM, Luikart G. Description and power analysis of two tests for detecting recent population bottlenecks from allele frequency data. Genetics. 1996;144:2001–2014. 8978083 10.1093/genetics/144.4.2001 PMC1207747

[30] Piry S, Luikart G, Cornuet JM. BOTTLENECK: a computer program for detecting recent reductions in the effective population size using allele frequency data. J Hered. 1999;90:502–503.

[31] Garza JC, Williamson EG. Detection of reduction in population size using data from microsatellite loci. Mol Ecol. 2001;10:305–318. 11298947 10.1046/j.1365-294x.2001.01190.x

[32] Paetkau D, Waits LP, Clarkson PL, Craighead L, Vyse E, Ward R, et al Variation in genetic diversity across the range of North American brown bears. Conserv Biol. 1998a;12:418–429.

[33] Fahrig L. Effects of habitat fragmentation on biodiversity. Annu Rev Ecol Evol Syst. 2003;34:487–515.

[34] Vaughan MR, Pelton MR. Black bears in North America. In: ET LaRoe III, Our Living Resources ed, Washington DC, USA; 1995. pp. 100–103.

[35] Scheick BK, McCown W. Geographic distribution of American black bears in North America. Ursus. 2014; 25:24–33.

[36] Lackey CW, Beckmann JP, Sedinger J. Bear historical ranges revisited: documenting the increase of a once-extirpated population in Nevada. J Wildl Manage. 2013;77:812–820.

[37] Paetkau D, Strobeck C. Microsatellite analysis of genetic variation in black bear populations. Mol Ecol. 1994;3:489–495. 7952329 10.1111/j.1365-294x.1994.tb00127.x

[38] Boersen MR, Clark JD, King TL. Estimating black bear population density and genetic diversity at Tensas River, Louisiana using microsatellite DNA markers. Wildl Soc Bull. 2003;31:197–207.

[39] Triant DA, Pace RM III, Stine M. Abundance, genetic diversity and conservation of Louisiana black bears (Ursus americanus luteolus) as detected through noninvasive sampling. Conserv Genet. 2004;5:647–659.

[40] Brown SK, Hull JM, Updike DR, Fain SR, Ernest HB. Black bear population genetics in California: signatures of population structure, competitive release, and historical translocation. J Mammal. 2009;90:1066–1074.

[41] Belant JL, Van Stappen JF, Paetkau D. American black bear population size and genetic diversity at Apostle Islands National Lakeshore. Ursus. 2005;16:85–92.

[42] Robinson SJ, Waits LP, Martin IB. Evaluating population structure of black bears on the Kenai Peninsula using mitochondrial and nuclear DNA analyses. J Mammal. 2007;88:1288–1299.

[43] Obbard ME, Howe EJ, Kyle CJ, Haselmayer J, Scheifley J. 2016. Estimating the abundance of American black bears (Ursus americanus) on the Bruce Peninsula. Science and Research Technical Report TR-13. Ontario Ministry of Natural Resources and Forestry, Peterborough, ON, Canada.

[44] Howe EJ, Obbard ME, Schaeffer JA. Extirpation risk of an isolated black bear population under different management scenarios. J Wildl Manage. 2007;71:603–612.

[45] Obbard ME, Coady MB, Pond BA, Schaefer JA, Burrows FG. A distance-based analysis of habitat selection by American black bears (Ursus americanus) on the Bruce Peninsula, Ontario, Canada. Can J Zool. 2010a;88:1063–1076.

[46] Pelletier A, Obbard ME, White BN, Doyle C, Kyle CJ. Small-scale genetic structure of American black bears illustrates potential postglacial recolonization routes. J Mammal. 2011;92:629–644.

[47] Suffling R, Clarke T, Evans M, Lamb L, May S, McKenzie I, et al Vegetation change and vegetation management in the Bruce Peninsula National Park and environs: final report. Waterloo: Faculty of Environmental Studies, University of Waterloo, ON, Canada; 1995. 135 pp.

[48] Howe EJ, Obbard ME, Kyle CJ, Selinger W, and Davis P. 2016. Estimated abundance of American black bears (Ursus americanus) on Cockburn Island, Ontario (Wildlife Management Unit 44) from barbed wire hair trap sampling. Science and Research Technical Report TR-04. Ontario Ministry of Natural Resources and Forestry, Peterborough, ON, Canada.

[49] Moreland P. Mammals of Bruce Peninsula National Park and Fathom Five National Marine Park, Resource description and analysis. Cornwall: Parks Canada, ON, Canada; 1996.

[50] Parks Canada. National Parks System Plan. 3rd ed. Gatineau: Parks Canada National Office, QC, Canada; 1997. 106 pp.

[51] Woods JG, Paetkau D, Lewis D, McLellan BN, Proctor M, Strobeck C. Genetic tagging of free-ranging black and brown bears. Wildl Soc Bull. 1999; 27:616–627.

[52] Keenan K, McGinnity P, Cross TF, Crozier WW, Prodöhl PA. diveRsity: an R package for the estimation of population genetics parameters and their associated errors. Methods Ecol Evol. 2013;4:782–788.

[53] Weir BS, Cockerham CC. Estimating F-statistics for the analysis of population structure. Evol. 1984;38(6): 1358–1370. 10.1111/j.1558-5646.1984.tb05657.x 28563791

[54] Jost L. GST and its relatives do not measure differentiation. Mol Ecol. 2008;17:4015–4026. 19238703 10.1111/j.1365-294x.2008.03887.x

[55] Nei M, Maruyama T, Chakraborty R. Bottleneck effect and genetic variability in populations. Evolution. 1975;29:1–10. 28563291 10.1111/j.1558-5646.1975.tb00807.x

[56] Queller DC, Goodnight KF. Estimating relatedness using genetic markers. Evolution. 1989; 43:258–275. 28568555 10.1111/j.1558-5646.1989.tb04226.x

[57] Krämer P, Gerlach G. Demerelate: Functions to calculate relatedness on diploid genetic data. R package version 0.8–0. 2013.

[58] Crow JF, Kimura M. An Introduction to Population Genetics Theory. New York: Harper and Row, NY, USA; 1970. 656 pp.

[59] Ohta T, Kimura M. A model of mutation appropriate to estimate the number of electrophoretically detectable alleles in a finite population. Genet Res Cambridge. 1973;22:201–204. 10.1017/s0016672300012994 4777279

[60] Waples RS, Do C. LDNe: a program for estimating effective population size from data on linkage disequilibrium. Mol Ecol Resources. 2008;8:753–756. 10.1111/j.1755-0998.2007.02061.x 21585883

[61] Tallmon D, Koyuk A, Luikart G, Beaumont M. ONeSAMP: a program to estimate effective population size using approximate Bayesian computation. Mol Ecol Resources. 2008;8:299–301. 10.1111/j.1471-8286.2007.01997.x 21585773

[62] Hill WG. Estimation of effective population size from data on linkage disequilibrium. Genet Res. 1981;38:209–216.

[63] Campton DE. Natural hybridisation and introgression in fishes: methods of detection and genetic interpretations In: Ryman N, Utter F, editors. Population Genetics and Fisheries Management. Seattle: University of Washington Press, USA; 1987 pp. 161–192.

[64] Bartley D, Bagley M, Gall G, Bentley B. Use of linkage disequilibrium data to estimate effective size of hatchery and natural fish populations. Conserv Biol. 1992;6:365–375.

[65] Waples RS, Do C. Linkage disequilibrium estimates of contemporary Ne using highly variable genetic markers: a largely untapped resource for applied conservation and evolution. Evol Applications. 2010;3:244–262. 10.1111/j.1752-4571.2009.00104.x PMC3352464 25567922

[66] Waples RS. A generalized approach for estimating effective population size from temporal changes in allele frequency. Genetics. 1989;121:379–391. 2731727 10.1093/genetics/121.2.379 PMC1203625

[67] Tallmon D, Luikart G, Beaumont M. Comparative evaluation of a new effective population size estimator based on approximate Bayesian computation. Genetics. 2004;167:977–988. 10.1534/genetics.103.026146 15238546 PMC1470910

[68] Waples RS. Genetic estimates of contemporary effective population size: to what time periods do the estimates apply? Mol Ecol. 2005;14:3335–3352. 10.1111/j.1365-294X.2005.02673.x 16156807

[69] Waples RS. A bias correction for estimates of effective population size based on linkage disequilibrium at unlinked gene loci. Conserv Genet. 2006;7:167–184.

[70] England PR, Cornuet JM, Berthier P, Tallmon DA, Luikart G. Estimating effective population size from linkage disequilibrium: severe bias in small samples. Conserv Genet. 2006;7:303–308.

[71] Peel D, Ovenden JR, Peel SL. NeEstimator: software for estimating effective population size, Version 13. Queensland Government, Department of Primary Industries and Fisheries; 2004.

[72] Berthier P, Beaumont MA, Cornuet JM, Luikart G. Likelihood-Based estimation of the effective population size using temporal changes in allele frequencies: a genealogical approach. Genetics. 2002;160:741–751. 11861575 10.1093/genetics/160.2.741 PMC1461962

[73] Peery MZ, Kirby R, Reid BN, Stoelting R, Doucet‐Bëer E, Robinson S, et al Reliability of genetic bottleneck tests for detecting recent population declines. Mol Ecol. 2012;21:3403–3418. 10.1111/j.1365-294X.2012.05635.x 22646281

[74] Di Rienzo A, Peterson A, Garza JC, Valdes AM, Slatkin M, Freimer NB. Mutational processes of simple-sequence repeat loci in human populations. Proc Natl Acad Sci USA. 1994;91:3166–3170. 8159720 10.1073/pnas.91.8.3166 PMC43536

[75] Weiser EL, Grueber CE, Jamieson IG. AlleleRetain: a program to assess management options for conserving allelic diversity in small, isolated populations. Mol Ecol Res. 2012;12(6):1161–1167. 10.1111/j.1755-0998.2012.03176.x 22925629

[76] Kolenosky GB. Reproductive biology of black bears in east-central Ontario. Int Conf Bear Res Manage. 1990;8:385–392.

[77] Obbard ME, Howe EJ. Demography of black bears in hunted and unhunted areas of the boreal forest of Ontario. J Wildl Manage. 2008;72:869–880.

[78] Sutton S. A study of black bear population dynamics and habitat usage within the great ecocystem of Bruce Peninsula National Park: field season report. Tobermory, ON, Canada; 1999.

[79] Lee DJ, Vaughan MR. Dispersal movements by subadult American black bears in Virginia. Ursus. 2003;14:162–170.

[80] Rogers LL. Effects of food supply and kinship on social behavior, movements, and population growth of black bears in Northeastern Minnesota. Wildl Monogr. 1987;97:3–72.

[81] Adams JM, Faure H. Palaeovegetation maps of the Earth during the last glacial maximum, and the early and mid-Holocene: an aid to archaeologists. J Archaeol Sci. 1997;24:623–647.

[82] Bouzat JL, Cheng HH, Lewin HA, Westemeier RL, Brawn JD, Paige KN. Genetic evaluation of a demographic bottleneck in the greater prairie chicken. Conserv Biol. 1998;12:836–843.

[83] Hoban S, Gaggiotti OE, Bertorelle G. The number of markers and samples needed for detecting bottlenecks under realistic scenarios, with and without recovery: a simulation-based study. Mol Ecol. 2013;22:3444–3450. 23967455 10.1111/mec.12258

[84] Chikhi L, Sousa VC, Luisi P, Goossens B, Beaumont MA. The confounding effects of population structure, genetic diversity and the sampling scheme on the detection and quantification of population size changes. Genetics. 2010;186:983–995. 10.1534/genetics.110.118661 20739713 PMC2975287

[85] McEachern MB, Van Vuren DH, Floyd CH, May B, Eadie JM. Bottlenecks and rescue effects in a fluctuating population of golden-mantled ground squirrels (Spermophilus lateralis). Conserv Genet. 2011;12:285–296.

[86] Sastre N, Vilà C, Salinas M, Bologov VV, Urios V, Sánchez A, et al Signatures of demographic bottlenecks in European wolf populations. Conserv Genet. 2011;12:701–712.

[87] Williamson-Natesan EG. Comparison of methods for detecting bottlenecks from microsatellite loci. Conserv Genet. 2005;6:551–562.

[88] Nei M. F-statistics and analysis of gene diversity in subdivided populations. Ann Hum Genet. 1977;41:225–233. 596830 10.1111/j.1469-1809.1977.tb01918.x

[89] Rees EE, Pond BA, Cullingham CI, Tinline RR, Ball D, Kyle CJ, et al Landscape modelling spatial bottlenecks: implications for raccoon rabies disease spread. Biol Letters. 2009;5:387–390. 10.1098/rsbl.2009.0094 PMC2679935 19324623

[90] Pimm SL, Dollar L Bass OL. The genetic rescue of the Florida panther. Anim Conserv. 2006;9:115–122.

[91] Fredrickson RJ, Siminski P, Woolf M, Hedrick PW. Genetic rescue and inbreeding depression in Mexican wolves. Proc R Soc London B.2007;274:2365–2371. 10.1098/rspb.2007.0785 PMC2288557 17609180

[92] Dunbar MR, Cunningham MW, Wooding JB, Roth RP. Cryptorchidism and delayed testicular descent in Florida black bears. J Wildl Diseases. 1996;32(4):661–664. 9359066 10.7589/0090-3558-32.4.661

[93] Stratman MR, Alden CD, Pelton MR, Sunquist ME. Long distance movement of a Florida black bear in the southeastern coastal plain. Ursus. 2001;12:55–58.

[94] Liley SG, Walker RN. Extreme movement by an American black bear in New Mexico and Colorado. Ursus. 2015;26(1):1–6.

[95] Clark JD, Huber D, Servheen C. Bear reintroductions: lessons and challenges: invited paper. Ursus. 2002;13:335–345.

[96] Wear BJ, Eastridge R, Clark JD. Factors affecting settling, survival, and viability of black bears reintroduced to Felsenthal National Wildlife Refuge, Arkansas. Wildl Soc Bull. 2005;33(4):1363–1374.

[97] Comly-Gericke LM, Vaughan MR. Survival and reproduction of translocated Virginia black bears. Bears: Their Biology and Management, Int Conf Bear Res Manage. 1997;9(2):113–117.

[98] Eastridge R, Clark JD. Evaluation of 2 soft-release techniques to reintroduce black bears. Wildl Soc Bull. 2001;29(4):1163–1174.

[99] Mills LS, Allendorf FW. The one-migrant-per-generation rule in conservation and management. Conserv Biol. 1996;10:1509–1518.

[100] Murphy SM, Cox JJ, Clark JD, Augustine BC, Hast JT, Gibbs D, et al Rapid growth and genetic diversity retention in an isolated reintroduced black bear population in the central appalachians. J Wildl Manage. 2015;79(5):807–818.

[101] Obbard ME, Howe EJ, Wall LL, Allison B, Black R, Davis P, et al Relationships among food availability, harvest, and human-bear conflict at landscape scales in Ontario. Ursus.2014;25:98–110.

[102] Hopkins JB III, Kalinowski ST. The fate of transported American black bears in Yosemite National Park. Ursus. 2013;24(2):120–126.

[103] Alldredge MW, Walsh DP, Sweanor LL, Davies RB, Trujillo A. Evaluation of translocation of black bears involved in human–bear conflicts in South‐central Colorado. Wildl Soc Bull. 2015;39(2):334–340.

[104] Growth Plan for Northern Ontario. Ontario Ministry of Municipal Affairs; 2011. 76 pp. Available: https://wwwplacestogrowca/images/pdfs/GPNO-finalpdf.

[105] Bouzat JL, Johnson JA, Toepfer JE, Simpson SA, Esker TL, Westemeier RL. Beyond the beneficial effects of translocations as an effective tool for the genetic restoration of isolated populations. Conserv Genet. 2009;10:191–201.

[106] Pienaar EF, Telesco D, Barrett S. Understanding people's willingness to implement measures to manage human–bear conflict in Florida. J Wildl Manage. 2015;79(5):798–806.

