# Supplementary material for: Determining causes of genetic isolation in a large carnivore (Ursus americanus) population to direct contemporary conservation measures
Source: PLoS One. 2017 Feb 24;12(2):e0172319. doi: 10.1371/journal.pone.0172319 (PMC5325280; doi:10.1371/journal.pone.0172319)
Supplement: S1 File — Simulations were performed in EASYPOP (Balloux 2001) and in BottleSim (Kuo and Janzen 2003), via the high-performance computer clusters offered on SHARCNET (www.sharcnet.ca). Results for all simulations are provided in S2 Table. (DOCX) [file pone.0172319.s001.docx]

**Supporting Information**

**S1 File. Justifications of parameters and procedures used in forward-time simulations.** Simulations were performed in EASYPOP [1] and in BottleSim [2], via the high-performance computer clusters offered on SHARCNET ([www.sharcnet.ca](http://www.sharcnet.ca)). Results for all simulations are provided in S2 Table.

*-****Historical migration***

We ran Scenarios 1 and 2 in EASYPOP [1] with 2 simulated populations respectively corresponding to black bears found on the Bruce Peninsula (BP) and in southeastern Ontario (SE-ON). SE-ON_SIM_ (simulated SE-ON population) was modeled as one panmictic population [3]. We used 2 alternative initial population sizes for each simulated BP population (BP_SIM_) based on black bear density levels from eastern Ontario (0.2 bear/km^2^; 0.4 bear/km^2^ [4]). Since the BP surface area is 1,100 km^2^, the population sizes extrapolated from these density estimates are N_BP_ = 220 and 440 [5]. These numbers encompass the latest population size estimates of BP black bears (N = 225-408 individuals [6]). The surface of bear habitat across SE-ON is about 11,000 km^2^. Based on the same extrapolation as for N_BP_, N_SE-ON_ = 2,200 and 4,400 individuals. Both populations included equal sex ratios based on data previously obtained in this region [5, 7].

For **Scenario 1**, we used 3 alternative migration processes between BP_SIM_ and SE-ON_SIM_:

1. a *null hypothesis* (50% of male migration; 50% of female migration). Here, we assumed that male migration was constrained by female migration, as males are not expected to disperse into habitat where females are absent [8, 9].
2. a *saturation hypothesis* (90% of male migration; 50% of female migration). Here, we modeled a high migration rate for both males and females. In brown bears, 24,000 km^2^ of suitable habitat can become fully occupied within 25 to 104 years [10]. Thus, we supposed that as free habitat was being colonized, latecomers were arriving in areas of high density, leading to high dispersal rates even in females to avoid competition for resources [11]. Although post-glaciation migration rates for males and females are unknown, we assumed a lower proportion of dispersing females than males due to the strong pattern of male-biased dispersal observed in mammalian species [12].
3. *a female philopatry* hypothesis (90% of male migration; 3% of female migration). Here, we assumed a situation close to what is currently observed in black bears, with high male dispersal and low female dispersal [13-17].

For **Scenario 2a,** we used various low parameters of migration to model genetic drift:

1. a *reduced level of movement hypothesis* (10% of male migration and 3% of female migration). We assumed a reduced migration rate for males compared to current estimates. Indeed, studies of black bears in New Mexico and Québec suggest that males can show reduced levels of migration under various density conditions [18, 19].
2. an *extremely reduced level of movement hypothesis* (3% of male migration and 3% of female migration). We assumed that male migration was as low as female migration. Although such a low level of male migration has not been reported in American black bear, when population density is well below carrying capacity, some males can stay within a 6 km distance of full siblings, parents, or offspring. [18].
3. *no migration*. We assumed that both males and females remained in their area of origin to obtain a reference for complete drift.

Under **Scenario 2b**, we further explored the parameter space of historical reduced migration around the one-migrant rule [20, 21] with:

1. a *0.1 migrant per generation hypothesis* (male migration only).
2. a *0.2 migrant per generation hypothesis* (male migration only).
3. a *0.5 migrant per generation hypothesis* (male migration only).
4. a *1 migrant per generation hypothesis* (male migration only).
5. a *2 migrants per generation hypothesis* (male migration only).
6. a *5 migrants per generation hypothesis* (male migration only).
7. a *10 migrants per generation hypothesis* (male migration only).

The number of alleles per locus in the Ontario black bear dataset from Pelletier et al. [3] varied from 10 to 27 (mean = 14.43). As such, we ran our simulations with 14 loci and 15 allelic states. We also used free recombination between loci, a mutation rate (µ) of 2*10^-4^ to fall within the mutation rate of mammals [22, 23], a single-step mutation model, and set our initial population at maximum genetic variability.

To run our model, our simulated panmictic population was required to have an initial genetic diversity similar to the one empirically observed in the SE-ON genetic cluster [3]. We used the results from the null hypothesis of Scenario 1 to check at which point in time the population reached a similar level of genetic variation. For N_SE-ON_ = 2,200 and 4,400, respectively, 1,000 and 3,000 generations were necessary for allelic richness and heterozygosity to reach a level comparable to what is currently observed in SE-ON. Based on this, each EASYPOP simulation was run for an additional 1,000 or 3,000 generations (with male and female migration set at 50% to simulate panmixia), before the rest of our simulation parameters were implemented.

We calculated one bear generation from demographic data recorded in a protected forested area located in southeastern Ontario (Algonquin Park – Obbard et al. unpublished data). It was calculated as the sum of the earliest reproduction age (5 years) and latest reproduction age (15 years) of females, divided by 2, giving a bear generation time of 10 years.

We simulated 100 replicates of Scenarios 1 and 2 under the 2 alternative density levels for 1,200, and 400 generations, for a total of 52 situations (13 migration rates * 2 population densities * 2 number of generations). The alternative number of generations enabled us to model bear colonization from the time at which vegetation returned to the Great Lakes region (12,000 years ago [24]), and from the time the Great Lakes region included mixed forests, while boreal vegetation was present in northern Canada (4,000 years ago [24]).

*-****Recent demographic bottleneck***

BottleSim [2] is a program that simulates population bottlenecks by implementing an overlapping generation model in which the user can vary population size over time. In BottleSim, we used our empirical data from SE-ON as our genotypic input file. This allowed the program to set the initial parameters of genetic diversity based on our previous study [3] to perform the analyses for **Scenario 3**.

In 1903 and 1908, large fires occurred on the BP as a consequence of agricultural and logging activities [25]. We presume that these fires, in addition to increased human presence, resulted in high direct and indirect mortality for BP black bears. Based on the amount of bear habitat damaged by each fire [25], we conducted 1,000 iterations of scenario 3 under alternative rates of population declines and recovery. We used 2 sudden drops in population size 5 years apart to reflect the occurrence of each fire. For the first drop, representing the smaller of the two fires, we used 3 alternative mortality proportions (10%, 20%, and 40%). For the second drop, which represented the larger fire, we used 90% and 70% mortality. These alternative mortality proportions allowed us to obtain results under both a conservative and a more drastic situation. We modeled population recovery (assumed to start following the first fire) through 2 alternative growth rates (a 10% growth rate, representing highly productive black bear populations in the Great Lakes St. Lawrence region, and a 5% growth rate, representing regulated, hunted populations [7]).

Black bear age distribution was estimated from survival and reproductive data collected in Ontario and on the BP (longevity: 20 years; age of reproductive maturation: 5 years; frequency of production of litters: once every 2 years [4, 7, 26]). Based on this age distribution, we estimated that the proportion of generation overlap was equal to 75%. As such, 75% of individuals were assigned a random age value, whereas the other 25% had their age set to zero in year 0. We used a dioecic reproductive system with random mating, and ran our simulations over a period of 120 years. We induced the first drop in population size at year 13^th^, and the second drop at year 18^th^ to represent the 1903 and 1908 fires. For comparison purposes, we also set null conditions by modeling a constant population size over the same period of time.

As the recovery time was not affected by the initial population size (N_BP_ = 220, 440) due to our use of a constant growth rate following the population decline, we only used a starting population size of 220 individuals for these simulations. In total, 13 situations were modeled under Scenario 3: 12 situations (3 initial mortality rates * 2 final mortality rates * 2 growth rates), and a situation representing null conditions (see Table A below).

**Table A. BottleSim parameters, and associated bottlenecked population sizes and recovery times based on an initial population size of 220 individuals.**

| Hypothesis | Mortality due to fire 1 | Mortality due to fire 2 | Growth rate | *Population size after fire 2* | *Recovery time (years) to initial population size* |
| --- | --- | --- | --- | --- | --- |
| Null | 0% | 0% | 1 | *220* | 0 |
| 1a | 10% | 90% | 1.05 | *22* | 53 |
| 1b | 10% | 90% | 1.1 | *22* | 30 |
| 2a | 20% | 90% | 1.05 | *21* | 53 |
| 2b | 20% | 90% | 1.1 | *22* | 30 |
| 3a | 40% | 90% | 1.05 | *16* | 59 |
| 3b | 40% | 90% | 1.1 | *19* | 31 |
| 4a | 10% | 70% | 1.05 | *66* | 30 |
| 4b | 10% | 70% | 1.1 | *66* | 18 |
| 5a | 20% | 70% | 1.05 | *64* | 31 |
| 5b | 20% | 70% | 1.1 | *66* | 18 |
| 6a | 40% | 70% | 1.05 | *48* | 37 |
| 6b | 40% | 70% | 1.1 | *58* | 19 |

*-****Reduced migration due to anthropogenic influences on the landscape***

**Scenario 4** corresponded to a low diversity due to highly reduced migration between SE-ON and BP as a consequence of human influences such as agriculture, industrial practices, and urban development over the last 400 years [27-29]. In EASYPOP, we used the same population and genetic parameters as for Scenarios 1 and 2, and simulated the following alternative migration rates between BP_SIM_ and SE-ON_SIM_:

1. a *0.1 migrant per generation hypothesis* (male migration only). This level of migration is extremely low and would not be high enough to maintain genetic diversity between 2 populations [30]. We investigated this value to obtain a deeper understanding of how fast differentiation could occur between 2 populations that have only diverged recently.
2. a *0.2 migrant per generation hypothesis* (male migration only). This level of migration is very low and would not be high enough to maintain genetic diversity between 2 populations [30]. We investigated this value to obtain a deeper understanding of how fast differentiation could occur between 2 populations that have only diverged recently.
3. a *0.5* *migrant per generation hypothesis* (male migration only). This level of migration is not considered high enough to maintain genetic diversity between 2 populations [30]. We are assuming that BP sites were part of the large continuum of Ontario black bear populations, and as such, they were historically as genetically diverse as SE-ON. Under these circumstances, such a low level of gene flow could explain the difference in diversity between BP and SE-ON.
4. a *1 migrant per generation hypothesis* (male migration only). This level of migration has been identified as the minimum at which negative effects of drift can be avoided [30]. However, a number of alleles as low as 2.25 and H_E_ as low as 0.27 can occur for populations that have only been isolated for a recent period of time and receive a similar number of migrants [31].
5. a *2 migrants per generation hypothesis* (male migration only). This level of migration corresponds to what has been observed between southeastern Ontario sites and BP sites in a previous study (*F*_ST_ = 0.127; N*m* = 1.7 [3]), based on N*m* = [(1/*F*_ST_)-1]/4 [32].
6. a *5 migrants per generation hypothesis* (male migration only). This level of migration represents the middle ground of the one-migrant rule proposed by Mills and Allendorf [21], and could maintain genetic variation depending on how reproductively successful the migrants are in the new population [33].
7. a *10 migrants per generation hypothesis* (male migration only). This level of migration has been suggested to maintain genetic variation between populations, even when the island model of migration is not fully respected [21]. Still, this number represents a lower amount of migrants than what is observed between southeastern and northwestern Ontario sites, where no landscape barrier to movement exists (*F*_ST_ = 0.013; N*m* = 18.5 [3]). As such, this level corresponds to reduced migration relative to the assumed historical rate.
8. *no migration*. We assumed that both males and females remained in their area of origin due to a complete barrier to movement induced by human activities between BP and SE-ON.

We ran 100 replicates of each hypothesis under the 2 alternative density levels for 40 generations, for a total of 16 situations (8 migration rates * 2 population densities * 1 number of generations).

*-****Reduced migration due to anthropogenic influences on the landscape and demographic bottleneck***

For each parameter set from Scenario 4 (n = 16), we used the EASYPOP output for which the heterozygosity and number of alleles were closest to the average (among the 100 iterations) as starting points to perform the simulations for **Scenario 5**. This allowed BottleSim to start with an initial genetic diversity representative of a reduced diversity resulting from a recent decrease in migration rate between BP and SE-ON. We used the same BottleSim parameters (n = 13) as for Scenario 3 to model the population decline and recovery, for a total of 208 situations.

**References**

[1] Balloux F. EASYPOP (Version 17): A computer program for population genetics simulations. J Hered. 2001;92:301-302.

[2] Kuo C, Janzen F. BottleSim: a bottleneck simulation program for long-lived species with overlapping generations. Mol Ecol Notes. 2003;3:669-673.

[3] Pelletier A, Obbard ME, Mills K, Howe EJ, Burrows FG, White BN, et al. Delineating genetic groupings in continuously distributed species across largely homogeneous landscapes: a study of American black bears (Ursus americanus) in Ontario, Canada. Can J Zool. 2012;90:999-1014.

[4] Yodzis P, Kolenosky G. A population dynamics model of black bears in Eastcentral Ontario. J Wildl Manage. 1986;50:602-612.

[5] Howe EJ, Obbard ME, Schaeffer JA. Extirpation risk of an isolated black bear population under different management scenarios. J Wildl Manage. 2007;71:603-612.

[6] Obbard ME, Howe EJ, Kyle CJ, Haselmayer J, Scheifley J. 2016. Estimating the abundance of American black bears (Ursus americanus) on the Bruce Peninsula. Science and Research Technical Report TR-13. Ontario Ministry of Natural Resources and Forestry, Peterborough, ON, Canada.

[7] Kolenosky GB. Reproductive biology of black bears in east-central Ontario. Int Conf Bear Res Manage. 1990;8:385-392.

[8] Klatt PH, Ritchison G. The effect of mate removal on the vocal behavior and movement patterns of male and female eastern screech-owls. Condor. 1994;96:485-493.

[9] Alberts SC, Altmann J. Balancing costs and opportunities: dispersal in male baboons. Am Nat. 1995;145:279-306.

[10] Pyare S, Cain S, Moody D, Schwartz C, Berger J. Carnivore recolonization: reality, possibility, and a non-equilibrium century for grizzly bears in the southern Yellowstone ecosystem. Anim Conserv. 2004;7:1-7.

[11] Clutton-Brock T-H, Lukas D. The evolution of social philopatry and dispersal in female mammals. Mol Ecol. 2012;21:472-492.

[12] Greenwood PJ. Mating systems, philopatry and dispersal in birds and mammals. Anim Behav. 1980;28:1140-1162.

[13] Rogers LL. Effects of food supply and kinship on social behavior, movements, and population growth of black bears in Northeastern Minnesota. Wildl Monogr. 1987;97:3-72.

[14] Schwartz CC, Franzmann AW. Dispersal and mortality rates of subadult black bears from the Kenai Peninsula, Alaska. J Wildl Manage. 1992;56:426-431.

[15] Lee DJ, Vaughan MR. Dispersal movements by subadult American black bears in Virginia. Ursus. 2003;14:162-170.

[16] Costello C. Estimates of dispersal and home-range fidelity in American black bears. J Mammal. 2010;91:116-121.

[17] Pelletier A, Obbard ME, White BN, Doyle C, Kyle CJ. Small-scale genetic structure of American black bears illustrates potential postglacial recolonization routes. J Mammal. 2011;92:629-644.

[18] Costello CM, Creel SR, Kalinowski ST, Vu NV, Quigley HB. Sex-biased natal dispersal and inbreeding avoidance in American black bears as revealed by spatial genetic analyses. Mol Ecol. 2008;17:4713-4723.

[19] Roy J, Yannic G, Côté SD, Bernatchez L. Negative density-dependent dispersal in the American black bear (Ursus americanus) revealed by noninvasive sampling and genotyping. Ecol Evol. 2012;2:525-537.

[20] Wright S. Evolution in mendelian populations. Genetics. 1931;16:97-259.

[21] Mills LS, Allendorf FW. The one-migrant-per-generation rule in conservation and management. Conserv Biol. 1996;10:1509-1518.

[22] Ellegren H. Mutation rates at porcine microsatellite loci. Mamm Genome. 1995;6:376-377.

[23] Crawford AM, Cuthbertson RB. Mutations in sheep microsatellites. Genome Res. 1996;6:876-879.

[24] Adams JM, Faure H. Palaeovegetation maps of the Earth during the last glacial maximum, and the early and mid-Holocene: an aid to archaeologists. J Archaeol Sci. 1997;24:623-647.

[25] Suffling R, Clarke T, Evans M, Lamb L, May S, McKenzie I, et al. Vegetation change and vegetation management in the Bruce Peninsula National Park and environs: final report. Waterloo: Faculty of Environmental Studies, University of Waterloo, ON, Canada; 1995. 135 pp.

[26] Obbard, ME, Howe, EJ. Demography of black bears in hunted and unhunted areas of the boreal forest of Ontario. J Wildl Manage. 2008;72:869-880.

[27] Butzer K. The Americas before and after 1492: an introduction to current geographical research. Ann Assoc Am Geogr. 1992;82:345-368.

[28] Muller MR, Middleton J. A Markov model of land-use change dynamics in the Niagara Region, Ontario, Canada. Landsc Ecol. 1994;9:151-157.

[29] Elliott KA. The forests of southern Ontario. For Chronicle. 1998;74:850-854.

[30] Wright S. Evolution in mendelian populations. Genetics. 1931;16:97-259.

[31] Dixon JD, Oli MK, Wooten MC, Eason TH, McCown JW, Cunningham MW. Genetic consequences of habitat fragmentation and loss: the case of the Florida black bear (Ursus americanus ﬂoridanus). Conserv Genet. 2007;8:455-464.

[32] Wright S. The genetical structure of populations. Ann Eugenics. 1951;15:323-354.

[33] Wang J. Application of the one‐migrant‐per‐generation rule to conservation and management. Conserv Biol. 2004;18(2):332-343.
